# Cessation of Bezafibrate in patients with chronic kidney disease improves renal function

**DOI:** 10.1038/s41598-020-76861-1

**Published:** 2020-11-13

**Authors:** Boris Zingerman, Danny Ziv, Netta Feder Krengel, Asher Korzets, Ilan Matok

**Affiliations:** 1 Community Nephrology Clinic, Clalit Health Services, Kiryat Ono, Israel; 2 Sackler School of Medicine, Tel Aviv University, Tel Aviv, Israel; 3 Department of Nephrology and Hypertension, Hasharon Hospital, Rabin Medical Center, 4941492 Petah Tikva, Israel; 4 Division of Clinical Pharmacy, Institute for Drug Research, School of Pharmacy, Faculty of Medicine, The Hebrew University of Jerusalem, Jerusalem, Israel

**Keywords:** Chronic kidney disease, Drug therapy, Dyslipidaemias

## Abstract

Bezafibrate (BzF) is eliminated by renal excretion and dosage must be reduced in patients with chronic kidney disease (CKD). There is a concern that BzF causes a further deterioration in renal function in patients with CKD. This study assessed whether BzF discontinuation or dose reduction in CKD patients improves renal function. 117 CKD patients treated with BzF between 2009 and 2014 were studied for demographics, comorbid conditions and laboratory variables. Data compared 2 groups: an intervention group of 64 patients where recommendations regarding BzF administration was implemented and a control group of 37 patients. Follow-up was maintained for 12 months. In the intervention group, estimated glomerular filtration rate (eGFR) increased from 38 to 42 mL/min/1.73 m^2^ (p = 0.01); blood urea levels decreased from 81 to 77 mg/dL (p = 0.04). Serum creatinine decreased by more than 0.2 mg/dL in 45% of the intervention group, as compared to 19% of the control group (p < 0.01). Improvement in eGFR was seen exclusively in patients who stopped BzF completely (eGFR increased from 38 to 44 mL/min/1.73 m^2^). In the intervention group, TG level increased from 183 to 220 mg/dL (p < 0.001). BzF cessation in approximately 50% of patients with CKD was associated with an increase in eGFR.

## Introduction

Hypertriglyceridemia is the primary lipid abnormality among chronic kidney disease (CKD) patients, with 40–50% of CKD patients having a fasting serum triglycerides (TG) level of > 200 mg/dL (2.26 mmol/L)^1,2^. Bezafibrate (BzF), a fibric acid derivative, can reduce serum triglyceride (TG) levels by up to 40–50%^3,4^. The usual dose of BzF is 200 mg immediate release (IR) three times a day or a 400 mg sustained release (SR) tablet once daily^5^. BzF excretion is nearly completely renal (95%), and it is therefore recommended to adjust the dose in CKD—as defined by an estimated glomerular filtration rate (eGFR) < 60 mL/min/1.73 m^2^ for at least 3 months^5,6^. Even at mild to moderate renal disease the SR formulation should be avoided, as plasma half life of the drug is markedly prolonged in these patients. At CKD—Stage IV, a dose of 200 mg every 24–48 h is recommended. In patients with CKD—Stage V, all BzF formulations should be avoided^4,5^. Indeed, the “Kidney Disease: Improving Global Outcomes (KDIGO)” guidelines regarding treatment of hypertriglyceridemia in CKD states that the use of fibric acid derivatives to reduce cardiovascular risk is not recommended. Fibrates may be considered only in a small number of CKD patients in which fasting TG levels > 1000 mg/dL, again—with the appropriate above-mentioned dose adjustments^7^.

The main concerns with BzF accumulation are kidney damage and rhabdomyolysis. BzF in patients with and without CKD, may cause an increase of serum creatinine (SCr)^8,9^. The joint appearance of both increased serum creatinine and urea levels imply a decreased eGFR—hopefully of a reversible nature. However in 2002, Hottelart et al. suggest that another fibrate, fenofibrate, causes a rise in both serum and urine creatinine levels via an increased production rate of creatinine, without any reduction in renal function^10^. The possible mechanisms responsible for possible fibrate-induced deterioration of renal function are unclear, yet several theories are available and include: (1) fibrates reduce the production of vasodilatory prostaglandins by inhibition of the enzyme cyclooxygenase-2^9^, with subsequent afferent glomerular blood vessel constriction, (2) fibrates can cause rhabdomyolysis-induced acute kidney injury, (3) BzF damages nephrons by injuring the proximal convoluted tubule^11^.

This study aimed to assess if BzF discontinuation or dose reduction in CKD patients could lead to an improvement in renal function, and also whether the serum lipid profile and blood glucose levels would be affected adversely by such BzF dose changes.

## Methods

### Study population

This retrospective study reviewed patients registered in the Dan–Petach Tikva District of “Clalit” Health Insurance Services, treated with BzF. The patients received BzF for at least 3 months before any intervention was carried out. A clinical pharmacist consultation regarding BzF dose adjustments because of the presence of an eGFR < 60 mL/min/1.73 m^2^ was given to family physicians between 2009 and 2014. Patients on maintenance dialysis therapy were excluded. Family physicians were allowed complete freedom as to what to do with their patients and their BzF dose. BzF purchases were monitored to ensure that BzF was no longer purchased or purchased at a reduced dose after the intervention, and that BzF purchases were continued unchanged in the control group. Follow-up was maintained for 12 months in both groups.

Data was extracted from the “Clalit” databases and included: age, gender, comorbid conditions, serum lipid profile, blood glucose, and creatine kinase (CPK) levels. In patients with TG levels > 300 mg/dL and who had no LDL-cholesterol levels in their records, the Friedewald formula was used to calculate the LDL-cholesterol level^12^**.** Calculation of eGFR was performed according to serum creatinine levels using the MDRD formula at MDRD.com^13,14^.

The study protocol was approved by the Clalit Health Services Internal Review Board—Helsinki Committee for Community Medicine. The study was conducted following the principles of the Declaration of Helsinki. Informed consent was waived by the ethics committee (Clalit Health Services Internal Review Board—Helsinki Committee for Community Medicine, COM—0013-15) for the entire study.

### Study design

We applied two study designs to assess the dose adjustment/discontinuation of BzF in patients with renal failure.

1. Case cross over study design. To initially assess the effect of discontinuation or dose adjustment of BzF on renal function, we chose all patients with renal failure that BzF was discontinued or its dose was adjusted. We followed these patients and compared in each patients the periods before the change and following the change to assess the discontinuation or dose adjustments on them.
2. A cohort study design was performed. Here, the patients in which BzF was discontinued or had a dose reduction were compared to patients in whom the BzF dosage was not changed.

### Potential confounders

The models were adjusted for universal confounders, variables known to be associated with the risk for renal failure and variables that might influence the choice of using BzF, including: age, gender, Charlson comorbidity index, diabetes mellitus and the type of intervention (BzF discontinuation or dose adjustment).

### Statistical analysis

Descriptive statistics was used to describe the study population. A univariate analysis was performed by using paired t-test in continuous variables that are normally distributed and Wilcoxon for non-normal distribution, to determine whether there was a statistically significant difference within the study groups from prior to post intervention. Independent t-test or Mann–Whitney U test were used to compare between groups. The χ^2^ test was used to compare differences in proportions (such as gender) between groups. Spearman’s correlation coefficient was used to evaluate the relationship between different parameters. p value < 0.05 was considered significant. IBM SPSS version 22 software was used^15^.

## Results

**Figure 1:**
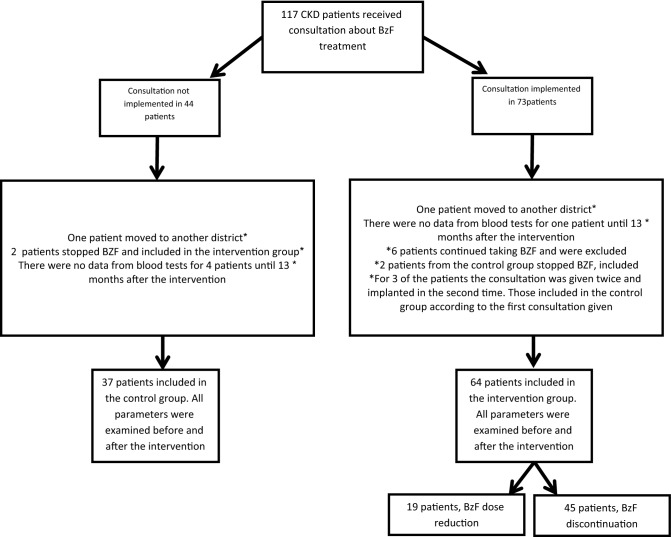
Study design.

One hundred and seventeen CKD patients were included in the initial recruitment, and 16 were excluded for various reasons (Fig. 1). 101 patients were included in the final data analysis; 64 patients in the intervention group, where the clinical pharmacist’s consultation was adhered to, and 37 patients who served as a control group. Baseline variables of the two groups are shown in Table 1. Patients’ age and baseline renal function were similar in both groups. There were more diabetic patients in the intervention group as compared to the control group [52 (81%) vs 12 (32%) patients respectively; p < 0.01]. This was unplanned, and simply a convenience sampling.

**Table 1:** Baseline Patients characteristics according to study groups.

Parameter/group	Intervention group	Control group	p-value
Number of patients	64	37	
Male (%)	42 (65%)	23 (62%)	NS
Diabetes (%)	52 (81%)	12 (32%)	< 0.01^†^
Age (years), mean ± SD	76 ± 9	75 ± 11	NS
Charlson index, mean ± SD	5 ± 2	4 ± 2	NS
Serum triglycerides level (mg/dL), mean ± SD	183 ± 83	210 ± 100	NS
Blood urea level (mg/dL), mean ± SD	81 ± 32	76 ± 25	NS
Blood glucose level (mg/dL), mean ± SD	132 ± 62	129 ± 57	NS
LDL-cholesterol (mg/dL), mean ± SD	85 ± 28	92 ± 24	NS
HDL-cholesterol (mg/dL), mean ± SD	42 ± 14	39 ± 14	NS
CPK (U/L), mean ± SD	108 ± 74	139 ± 147	NS
Serum creatinine level (mg/dL), mean ± SD	1.78 ± 0.6	1.69 ± 0.5	NS
eGFR (mL/min/1.73m^2^), mean ± SD	37.8 ± 1.4	38.6 ± 1.8	NS

*HDL* high density lipoprotein, *LDL* low density lipoprotein, *eGFR* estimated glomerular filtration rate, *CPK* creatine kinase.

^†^Chi squared.

### Serum creatinine and estimated glomerular filtration rate, blood urea levels

**Figure 2:**
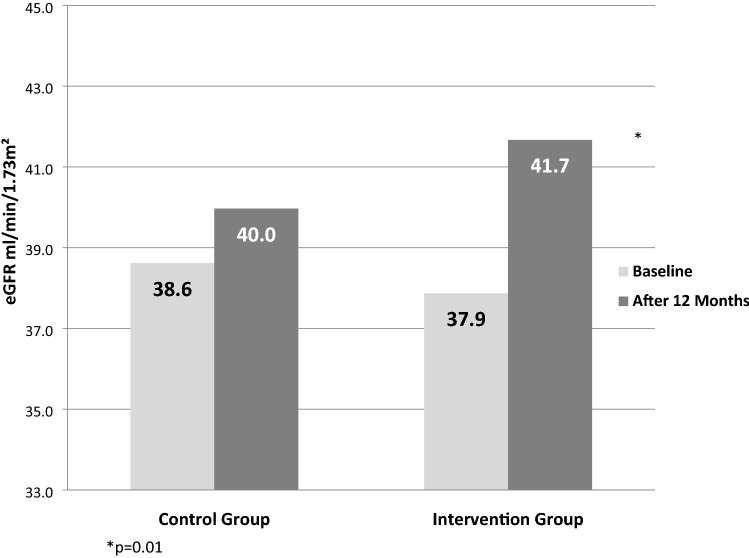
eGFR changes in both groups.

**Figure 3:**
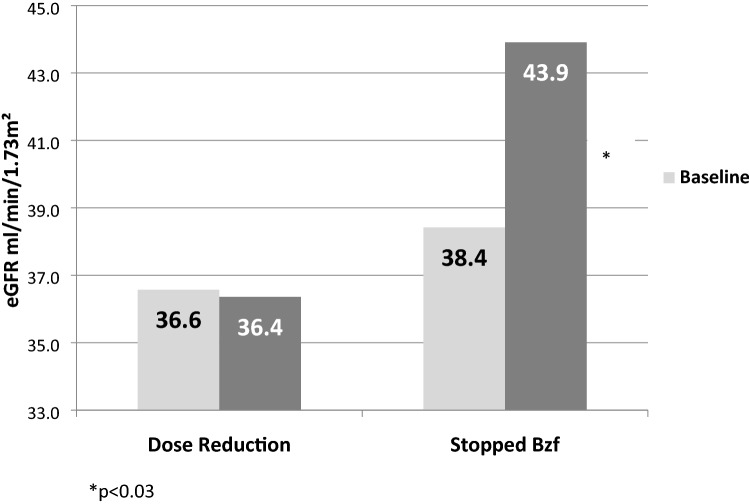
eGFR changes according to intervention type.

A decrease in serum creatinine and an increase in eGFR was observed in both groups, although the changes were significant only in the intervention group where eGFR rose from 38 to 42 mL/min/1.73 m^2^ (p = 0.01; Fig. 2). However, this improvement was seen nearly exclusively only in patients who stopped BzF (45/64 patients). In these patients eGFR increased from 38 to 44 mL/min/1.73 m^2^ (p = 0.03; Fig. 3).

Twenty nine patients (45%) of the intervention group had a decrease in serum creatinine ≥ 0.2 mg/dl compared to 7 (19%) patients in the control group (p < 0.01). In this subgroup of 29 patients—serum creatinine level at baseline was 1.8 ± 0.5 mg/dL and 1.4 ± 0.4 mg/dL at the end of the study. Of these 29 patients—20 (69%) patients were males, 22 (76%) patients were diabetics and 19 (66%) patients were treated with angiotensin converting enzyme inhibitors (ACEi) or angiotensin receptor blockers (ARB). Twenty one (71%) of these patients had BzF stopped as their intervention.

Serum urea levels decreased in the intervention group: 81 to 77 mg/dL (p = 0.04). This change was correlated to the decrease in serum creatinine. Before the intervention the correlation between blood urea and serum creatinine was r = 0.62; after the intervention a correlation of r = 0.54 was found.

### Lipid profile

Serum TG level increased in the intervention group from 183 to 221 mg/dL (p < 0.01). In patients who stopped BzF-serum triglycerides level increased from 161 to 220 (p < 0.01). One patient in the intervention group had a serum TG level > 500 mg/dL at study end. All changes in the lipid profile of the control group were insignificant (Table 2).

**Table 2:** Comparison between baseline and end of study in both groups.

Parameter	Control group			Intervention group		
	Baseline	At end of study	p-value	Baseline	At end of study	p-value
Serum creatinine (mg/dL), mean ± SD	1.69 ± 0.5	1.66 ± 0.5	0.33^†^	1.78 ± 0.6	1.75 ± 0.9	0.02^†^
eGFR (ml/min/1.73 m^2^), mean ± SD	38.6 ± 11.4	39.9 ± 14.1	0.24^†^	37.8 ± 11.2	41.6 ± 15	0.01*
Blood urea (mg/dL), mean ± SD	76 ± 25	74 ± 22	0.74*	81 ± 32	77 ± 39	0.04^†^
Triglycerides (mg/dL), mean ± SD	210 ± 100	203 ± 77	0.48^†^	183 ± 83	221 ± 95	< 0.01^†^
LDL-cholesterol (mg/dL), mean ± SD	92 ± 24	84 ± 26	0.08^†^	85 ± 28	83 ± 33	0.45^†^
HDL-cholesterol (mg/dL), mean ± SD	39 ± 14	36 ± 11	0.30*	42 ± 14	40 ± 11	0.03^†^
Blood glucose (mg/dL), mean ± SD	129 ± 57	141 ± 78	0.77^†^	132 ± 62	146 ± 68	0.01^†^
CPK (U/L), mean ± SD	139 ± 147	73 ± 47	0.03^†^	108 ± 73	137 ± 240	0.01^†^

*HDL* high density lipoprotein, *LDL* low density lipoprotein, *eGFR* estimated glomerular filtration rate, *CPK* creatine kinase.

*Paired sample t-test.

^†^Wilcoxon.

### Blood glucose levels

Blood glucose levels increased in the intervention group from 132 to 146 mg/dL (p = 0.01; Table 2). For those patients in whom BzF was discontinued, blood glucose levels increased from 130 to 151 mg/dL (p = 0.01). The control group did not have any significant changes in blood glucose levels (Table 2). The increase in blood glucose levels was significant only for those patients with underlying diabetes mellitus, as blood glucose increased from 138 to 151 mg/dL (p = 0.04).

### Creatine kinase (CPK) levels

No significant changes in CPK were found in the intervention group (Table 2). In the control group a significant decrease (139 to 73 U/L, p = 0.03; Table 2) in CPK was observed.

## Discussion

Hypertriglyceridemia is common. In the United States, the National Health and Nutrition Examination Surveys (NHANES) from 1999 to 2004 found that the percentage of adults with significantly high serum triglyceride levels above 500 mg/dL was ~ 2%^16^.

Elevated TG levels are independently associated with an increased risk of cardiovascular events. The Veterans Affairs High-Density Lipoprotein Intervention Trial (VA-HIT) evaluated the effect of gemfibrozil in patients with established coronary heart disease^17^. Within this cohort of ~ 2500 patients, there were ~ 1000 patients with impaired renal function: ~ 600 and 400 patients with creatinine clearances of 61 to 75 and 30 to 60 mL/min respectively. Among the patients with CKD, gemfibrozil therapy lowered the risk of the primary endpoint of coronary death and nonfatal myocardial infarction, but had no effect on total mortality. On the other hand, gemfibrozil caused a significant decline in renal function with ~ 6% of gemfibrozil treated patients experiencing a sustained increase in serum creatinine values. In the ACCORD Lipid trial, fenofibrate showed no overall benefit when added to a statin in patients with type 2 diabetes, but “tended” to improve outcomes in the subset of patients with both elevated triglyceride levels and low HDL-C levels^18^.

This study contributes to the evidence that BzF might have a reversible renal damage effect among CKD patients. Using eGFR, as assessed by serum creatinine levels, renal function improved in the intervention group in this study, and this was obviously evident in patients who had their BzF stopped completely. In nearly half the patients involved in the intervention (including some with a dose reduction only), a decrease in serum creatinine level ≥ 0.2 mg/dL was seen and such an improvement may be of clinical significant for CKD patients^19,20^. Importantly, the improvement in renal function associated with the change in BzF dosage was common in diabetic patients, most of whom were receiving ACEi or ARB. It seems that diabetics with CKD may benefit from an intervention regarding BzF therapy.

Previous studies have demonstrated changes in serum creatinine levels related to changes in BzF dose, but without an effect on eGFR. This may actually indicate a rise in serum creatinine levels after BzF is commenced but without any real effect in renal function^10^. However, as with the VA–HIT study, other studies have shown small but statistically significant and probably clinically relevant progressive increases in serum urea and creatinine levels after initiation of fibrate therapy in patients with normal renal function and in patients with CKD, and this is regarded as a probable class effect^17^. Both Broeders et al. and Charach et al. showed that changes in BzF dose led to decreases in serum urea levels, implying improvement in renal function; while Charach et al. demonstrated a decrease in serum creatinine ≥ 0.2 mg/dL in ~ 60% of their intervention group^8,9^. A true, but reversible, deterioration in renal function can possibly be explained by changes in the production of vasodilatory prostaglandins and by a proximal tubule damage, which was clinically reversible in 3 renal transplant patients after cessation of BzF (no repeat renal biopsies were performed in these patients)^9,11^. Serum cystatin and homocysteine levels, molecules excreted by the kidneys, rise after initiation of fibrate therapy, suggesting that fibrates cause a genuine reduction in eGFR^9^.

As expected, serum TG levels rose significantly in the intervention group. However during the follow-up period of 1 year, serum TG levels > 1000 mg/dL was not seen in any patient, this being the level needed to justify fibrate therapy for CKD patients^7^. Only one patient had a serum TG level > 500 mg/dL, a level in which fibrate therapy is to be considered according to the Israeli guidelines for treatment of hypertriglyceremia^21^. Thus, the initial need of BzF among the studied patients may be questionable. Even so, the mild increase in serum TG levels may be considered “bearable” as it was accompanied by an improvement in renal function, a point of undoubted importance in CKD patients, especially as renal insufficiency has now been shown to be an independent predictor of cardiovascular events and mortality among individuals with coronary disease or multiple cardiovascular risk factors^22,23^.

In diabetic patients, BzF cessation led to a significant rise in blood glucose levels. This effect of BzF on blood glucose levels is important both in the diagnosis and treatment of diabetic patients, as their blood glucose levels should be closely monitored as changes inBzF therapy are made^24^.

We failed to show a significant decrease of CPK in the intervention group. This data, regarding CPK levels in patients treated with fibrates is consistent with other studies that did not show significant CPK changes associated with fibrates when they were given without concomitant statin therapy^9,24^.

This study has several limitations. Firstly, other medications, apart from BzF, that could affect renal function were not taken into account during this study^25^. Secondly, eGFR was calculated solely using the MDRD equation without relying on a more accurate measurement of GFR using 24 h urine collections. Due to lack of data regarding the weight of some participants, this study did not use the Cockcroft-Gault equation. Importantly, the decrease in the serum creatinine level was accompanied by a co-related decrease in blood urea levels, and this fact adds strength to the assumption that BzF may have a harmful effect on renal function in patients with CKD, and that this damage may be reversible.

To conclude, cessation of BzF treatment has a positive effect on renal function in CKD patients as shown by the improvement of eGFR and decrease in serum creatinine in early half of the intervention group. Dose reduction in patients with chronic kidney disease may be insufficient to negate the effects of BzF on the kidneys, and therefore the correct therapeutic decision with a possible BzF-induced reduction in renal function should be to stop the drug altogether. Stopping BzF had a negative effect on serum TG levels, and on blood sugar levels in diabetic patients.
